# Development and evaluation of a mechanical chest compression device for standardized rodent cardiopulmonary resuscitation

**DOI:** 10.1038/s41598-025-31959-2

**Published:** 2025-12-08

**Authors:** Sam Joé Brixius, Jakob Wollborn, Johannes Dinkelaker, Sonja Bröer, Rita Sanchez-Brandelik, Katharina Denzer, Martin Czerny, Georg Trummer, Felix Particius Hans, Hans-Jörg Busch, Jan-Steffen Pooth

**Affiliations:** 1 Department of Cardiovascular Surgery, Medical Center, Medical Faculty, University Heart Center Freiburg-Bad Krozingen, University of Freiburg, Hugstetter Strasse 55, 79106 Freiburg, Germany; 2 Department of Anesthesiology, Perioperative and Pain Medicine, Brigham and Women’s Hospital, Harvard Medical School, Boston, USA; 3 Center for Experimental Models and Transgenic Service, Medical Center, Medical Faculty, University of Freiburg, University of Freiburg, Stefan-Meier- Strasse 17, 79104 Freiburg, Germany; 4 Institute of Pharmacology and Toxicology, School of Veterinary Medicine, Freie Universität Berlin, Berlin, Germany; 5 Faculty of Medicine, University of Freiburg, 79085 Freiburg, Germany; 6 University Emergency Department, Medical Center, Medical Faculty, University of Freiburg, University of Freiburg, Hugstetter Strasse 55, 79106 Freiburg, Germany

**Keywords:** Cardiopulmonary resuscitation, Chest compression, Mechanical chest compression device, Rats, Reperfusion, Rodent animal model, Ventricular fibrillation, Arrythmia, Emergency care medicine, Experimental models of disease, Preclinical research, Heart failure, Translational research, Cardiac device therapy

## Abstract

Small animal models are indispensable in cardiopulmonary resuscitation (CPR) research. High-quality CPR, characterized by consistent chest compression rate, depth, and positioning is crucial for survival. However, achieving standardization in manual high-frequency chest compressions in small animal models remains technically challenging. This study evaluated the reproducibility of manual chest compressions and introduced a novel mechanical chest compression device (MCD) designed to improve consistency in rodent experiments. In an in vitro setup, manual compressions were performed by ten participants at target rates ranging from 100 to 260 bpm, guided by a metronome. Compressions performed on a fluid-filled polymer reservoir were analyzed for the compression rate, variability, and time within a ± 10% target range. A color indicator was used to assess the variability of the compression point. A small animal MCD was designed and tested under the same conditions. In vivo, 5 Sprague-Dawley rats underwent 5 min of electrically induced normothermic cardiac arrest followed by 8 min of external chest compressions using the MCD. Obtained data was compared to the in vitro results. A total of 21,650 manual and 20,098 mechanical compressions were analyzed. At 200 bpm, chest compressions using the MCD were significantly more precise (201 ± 1.2 bpm) than manual compressions (218 ± 21 bpm, *p* < 0.001) with a significant reduced compression point variability (1.7 ± 0.1 cm^2^ vs. 10.8 ± 3.1 cm^2^, *p* < 0.001). Manual compressions maintained target rate in 58.8% of time compared to 100% for the MCD. In vivo testing confirmed these findings with chest compressions remaining within the target range 100% of the time and showing minimal rate variability (1.8 ± 1.7 bpm). These results highlight the limitations of manual chest compressions and demonstrate the potential of the MCD to enhance standardization and reproducibility in rodent CPR research.

**Supplementary Information:**

The online version contains supplementary material available at 10.1038/s41598-025-31959-2.

## Introduction

Cardiac arrest remains one of the leading causes of death worldwide^1^. Rapid and high-quality cardiopulmonary resuscitation (CPR) is a key determinant of survival, making consistent chest compression rate, depth, and accurate positioning of the compression point critically important^2,3^. Further research is urgently needed to better understand ischemia-reperfusion injury, enhance post-resuscitation care, and explore emerging fields such as extracorporeal resuscitation. In experimental research, small animal models continue to play an essential role^4^.

In line with ethical considerations and recognizing the advantages of small animal models —such as greater availability of standardized animal models — approximately 45% of scientific groups involved in CPR research utilize them^4^. Maintaining the consistency and effectiveness of chest compressions is crucial in both clinical and preclinical research settings to optimize patient outcomes and ensure reliable scientific data. Although manual chest compressions are widely utilized in small animal models, they are inherently variable due to human factors such as fatigue, inconsistencies in technique and inter-operator variation, although this was not demonstrated in any published work to date.

External chest compressions in small animal models are typically performed manually with an acoustic metronome at rates of 200–400 bpm^5,6^. However, standardizing high-frequency manual chest compressions in small animal models presents a significant challenge. To address this, some research groups have adopted mechanical chest compression devices (MCDs) to ensure consistent CPR quality across experiments. The designs of these MCDs vary widely, including devices such as the rat thumper^7^, pneumatically driven compressors^8^, pneumatic pistons^9^, and even repurposed sewing machines^10^. In many cases, studies do not provide detailed descriptions of the custom-made devices used.

While standardized MCDs have been established for human and large animal models showing superior blood pressure, blood flow and ROSC rates^11–14^, no standardized MCD has been developed, validated and published specifically for small animal models. Therefore, our study aimed to develop a mechanical chest compression device to enable standardized, high-quality CPR in rats and to evaluate its potential benefits compared to manual chest compressions.

## Materials and methods

### Development of mechanical chest compression device

The MCD was drafted using the computer-aided design (CAD) software Fusion 360 (Autodesk, USA), and computer-based simulations were used to define the component requirements and design details (included as Additional file 1). The majority of components were produced using 3D printing technologies based on fused deposition modelling (FDM) and PLA filament (polylactic acid filament).

**Fig. 1:**
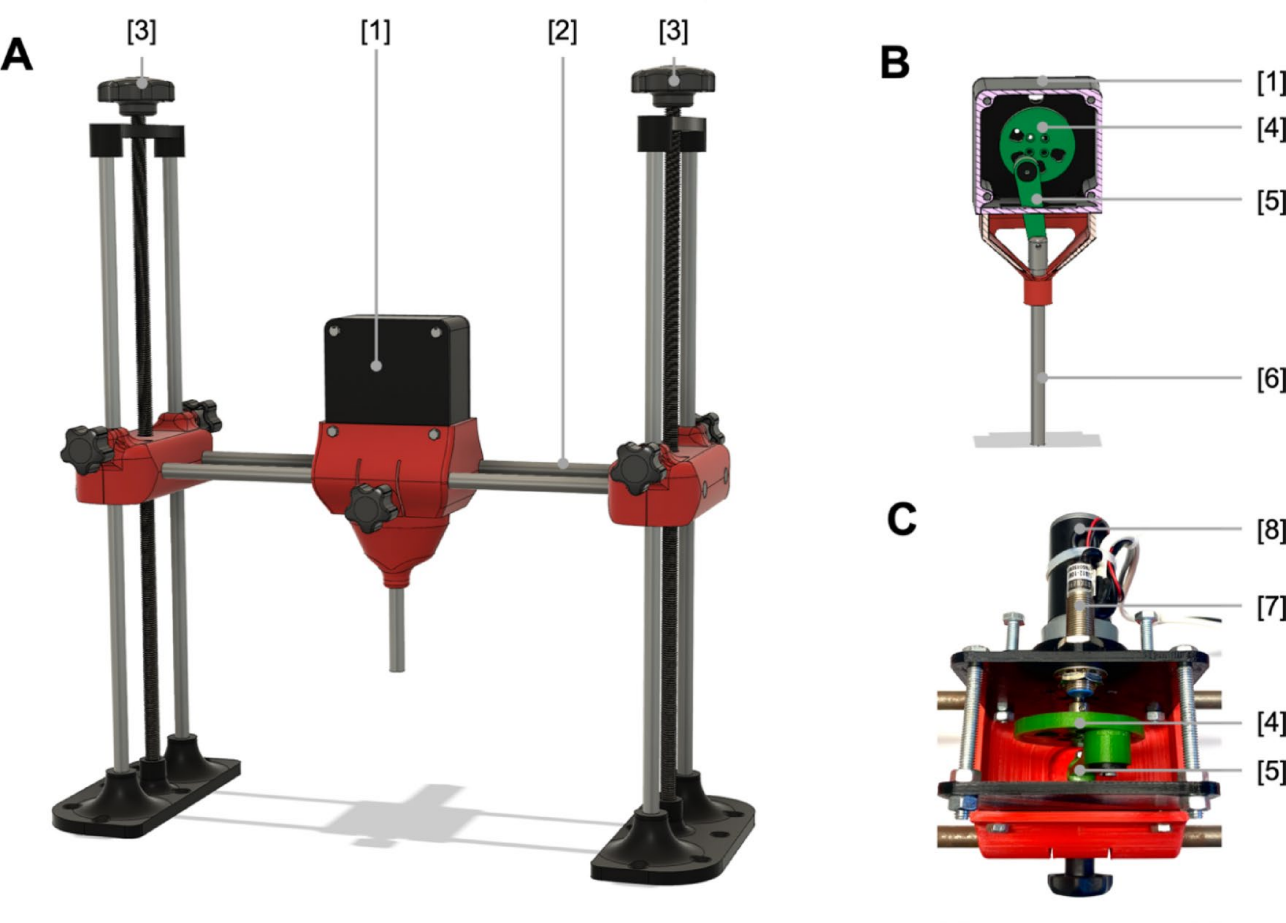
Illustration of mechanical chest compression device for small animal models (**A**) Computer-aided design (CAD) draft of the mechanical resuscitation device^1^. Housing containing the mechanic components^2^ Horizontal axis^3^ Vertical axis with central threated rod for height adjustment (**B**) Mechanical design with eccentrically loaded piston connected to an electric motor^1^. Housing^4^ Flywheel^5^ Connecting rod^6^ Aluminum piston. (**C**) Top view picture on mechanic components with magnetic inductive sensor facing towards the flywheel controlling the actual rotation speed^7^. Inductive sensor^8^ Electric motor. The production files and animation of the device are provided in the additional files.

The first prototype of the MCD comprised of a height-adjustable horizontal axis to which a housing - containing the mechanical components - was mounted (see Fig. 1 and Additional file 2). This allowed for the precise positioning of the selected compression point along the horizontal axis. The horizontal axis was attached to two vertical axes, each consisting of two steel rods measuring 12 mm in diameter and a metric threaded rod (M12), which enabled the horizontal axis to be adjusted in height. The piston’s lower vertical positioning can be modified between 0 mm and 150 mm in relation to the underlying surface, thereby enabling the apparatus to be adapted for use with animals of varying sizes.

The interior of the housing contains a 60 mm flywheel, which is driven by a 12 V brush motor and transmits the resulting rotational force to a 12 mm aluminum piston through an eccentrically mounted connecting rod. An adjustable motor driver controls the motor, and the power applied is visualized on an LCD display. Moreover, the actual compression rate is subjected to continuous monitoring through the use of a magnetically inductive sensor, which is mounted within the housing facing towards the flywheel. The magnetic equivalent is affixed to the outer radius of the flywheel and traverses the sensor with each rotation.

The design allows for a maximum possible compression depth of 38 mm at a range of supported compression rate of 100–400 bpm. The compression rate can be adjusted continuously via potentiometer. By utilizing a flywheel-driven piston in this first version of MCD, the compression waveform was fixed to a sinusoidal waveform that cannot be modified. The compression-to-decompression ratio therefor was fixed to be 50:50. With a total size of 51 × 45 × 22 cm (W x H x D) and a weight of 10 kg, the MCD can freely be repositioned and the compression point can be varied as required. The actual compression rate is displayed on the LCD display in real time.

### In vitro study

In order to reduce the necessity for animal testing and to comply with the 3R principle^15^, a proof-of-concept study was conducted using a novel in vitro set-up. The primary objective was to investigate the intra- and inter-provider variability of manual chest compressions compared to the new mechanical chest compression device (MCD). In both cases, chest compressions were performed on a fluid-filled, flexible and mechanically robust polymer reservoir. The actual compression rate was measured by recording pressure changes in the reservoir. The pressure measuring system and the polymer reservoir were filled with saline at room temperature. Data was recorded with a sampling rate of 1000 Hz using a pressure transducer (Physiological Pressure Transducer, Clip-on BP Domes, ADInstruments, Oxford, UK) and Powerlab C software (ADInstruments, Oxford, UK).

*N* = 10 participants with prior experience in resuscitation and resuscitation research were recruited to generate data on manual chest compressions. First, the participants were instructed to perform compressions with a constant compression rate of 200 bpm over a period of 10 min with the help of an acoustic metronome in the test setup described before. After a break of at least 10 min, the target compression rate and metronome were set to 100 bpm, and the participants were instructed to maintain the target compression rate for a period of one minute each at increasing rates up to 260 bpm (100, 120, 140, 160, 180, 200, 220, 240 and 260 bpm). Each change in compression rate was announced by the investigator immediately after the preceding acquisition period ended. Following each rate adjustment, participants were given a 10-second familiarization period before data recording commenced. To ensure the highest possible degree of standardization in manual compression, the acoustic metronome was adjusted accordingly in all cases.

The observational nature of this component of the study, in combination with the absence of any personal or identifiable data, resulted in the waiver of ethical approval and informed consent by the local ethic committee based on national law (Ethics Committee Albert-Ludwigs-University of Freiburg, § 15 BO LÄK BW, Request Number 25-1277). Members of the laboratory and the scientific working group were invited to participate as participants. It is not possible to trace the data back to individual participants, and the research was conducted in accordance with the relevant guidelines.

Mechanical chest compressions were assessed by using a prototype of our MCD in the same way as described above. Again, 10 min with a constant frequency of 100 bpm followed by one-minute intervals at increasing target frequencies (20 bpm steps from 100 to 260 bpm) were analyzed. A total of 10 runs were performed and analyzed. The compression piston was first levelled to the surface of the polymer reservoir. The compression rate was set to the target rate and the measurement started. The actual rate was continuously monitored using the build-in sensor. A deviation of ± 10% was tolerated without readjustment of the set frequency. Data acquired during the adjustment period of the motor speed were excluded from further analysis.

In both setups, a color indicator was used to check for compression point variability. The indicator was applied to the participants fingers or the piston, and the compressions were applied on an absorbent detector surface. The detector surface was then digitalized and analyzed using an image editing software (ImageJ, Version 2.0.0). To account for the different baseline compression areas of the participants’ fingers or the piston of the device, the total area was corrected to the baseline area by calculating a ratio.

### In vivo study

The animal study protocol was approved by the local animal welfare and licensing committee of the city of Freiburg, Germany, Regierungspräsidium Freiburg (G-21/050). The research was conducted in accordance with the directive 2010/63/EU of the European Parliament and the Council and the ARRIVE guidelines.

### Experimental animals

The experimental design and handling of animals were based on a previously published model^16^. *N* = 5 adult Sprague-Dawley rats of both sexes (530–630 g) were included in this study to validate the observations from the previous in vitro experiments. Both, male and female rats were used, as no sex-dependent differences in chest compressions have been proven so far in rats. Standard chow and tap water were provided *ad libitum* until the start of the experiment.

### Animal preparation

Following inhalation of 5% isoflurane (Piramal Critical Care B. V., Voorschoten, Niederlande) and subcutaneous injection of carprofen (Rimadyl^®^, Zoetis, Berlin, Germany 5 mg/kgBW (bodyweight)) the animals were monitored using a three-lead electrocardiogram (ECG). The animals were placed on a heated small animal operating table (Medax, Neumünster, Germany), a 5 Fr. rectal temperature probe was placed and the baseline body temperature was held constantly at 37.0 ± 1 °C.

Using aseptic techniques, a PE-50 catheter was placed in the right femoral vein for further fluid and drug administration. Anesthesia was maintained intravenously by infusion of midazolam and fentanyl (midazolam: 7 mg/kgBW/h and fentanyl: 70 µg/kgBW/h, Panpharma GmbH, Trittau, Germany), and isoflurane was suspended. Balanced crystalloid solution (Ringer-Infusionslösung, B.Braun, Melsungen, Germany, 10 mL/kgBW/h) was used as primary fluid substitution to maintain euvolemia. For invasive blood pressure monitoring, a PE-50 catheter was placed in the left carotid artery. All catheters were flushed with balanced crystalloid solution containing 10 I.E./ml heparin (B.Braun, Melsungen, Germany) before insertion. Animals were tracheostomized with a 14-gauge cannula and ventilated volume-controlled (FiO2 0.3, tidal volume 8–10 ml/kgBW, inspiration to expiration ratio 1:2, ventilation rate 40–50/min, PEEP 5). A bolus of 1 mg rocuronium was given before baseline measurements to prevent grasping.

### Experimental protocol

Cardiac arrest (CA) was induced transesophageally using a 5 Fr. pacing catheter (Edwards Swan-Ganz Bipolar Pacing Catheter 1,3 ml Cap D97120F5) and electric current at 50 Hz/5 mA (Fibrillator Fi 10 M, Stöckert, München) over 2 min. Once mean arterial pressure (MAP) dropped below 25 mmHg, CA was left untreated for 5 min. For this time, the pacing catheter was removed, the orotracheal tube disconnected from the ventilator, the intravenous anesthesia discontinued and the heat blanket turned off. The MCD was positioned with its stamp 0.5 cm superior the xiphoid in mid-thoracal line. After 5 min of untreated CA, ventilation was resumed with FiO2 of 1.0 with the previous settings and the MCD was switched on at a target rate of 200 bpm for the next 8 min. During chest compressions, no electrical or pharmacological defibrillation attempts were made in order to simulate refractory CA. At the conclusion of the experiment, animals were euthanized under deep anesthesia by intravenous administration of 1.5 mL potassium chloride (KCl) solution (7.45%).

### Data analysis

Data acquisition and analysis were realized using the software Labchart Pro (ADInstruments, Oxford, UK, Version 8.1.25). The pressure data was recorded at 1000 Hz and analyzed using the peak analysis tool (ADInstruments, Oxford, UK, Version 8.1.25) providing automatic detection of the multiple signal peaks. The detected peaks were manually validated by the same investigator. The actual compression rate was calculated for each compression by determining the time interval between two consecutive pressure peaks (Δt). The reciprocal of this interval (1 / Δt) was multiplied by 60 to obtain the instantaneous compression rate in beats per minute (bpm). The total number of compressions, mean, minimal and maximal compression rate were determined. Variance and standard deviation (SD) of compression rates of all analyzed compressions were calculated. In order to correct for different mean compression rates between booth groups, the coefficient of variance was obtained by dividing SD by mean compression rate. Time, in which the actual compression rate was within a range of ± 10% of the target compression rate, was assessed and expressed as a fraction of the total time. A ± 10% tolerance threshold was chosen to account for minor signal fluctuations due to sensor response and sampling limitations, aligning with the ± 10% rate deviation accepted in current ALS guidelines (100–120 bpm)^3^.

### Statistical analysis

Statistical analysis and visualization were performed using R statistical software (version 4.2.3)^17,18^. Tests for normal distribution by Q-Q plot and Shapiro–Wilk test were performed. Data are presented as mean value with SD. Differences between groups were compared applying Mann–Whitney rank sum test or student t-Test as appropriate. Statistical analyses regarding serial measurements were conducted in a mixed-effects model using the R packages lme4 and lmerTest^19^. The interaction over time was tested in a simple linear model. A significance level of 5% was chosen and confidence intervals of 95% were reported.

## Results

### In vitro evaluation

As part of this study, a total of 41.748 compressions were analyzed (21.650 manual compressions and 20.098 mechanical compressions in the in vitro setup).

**Fig. 2:**
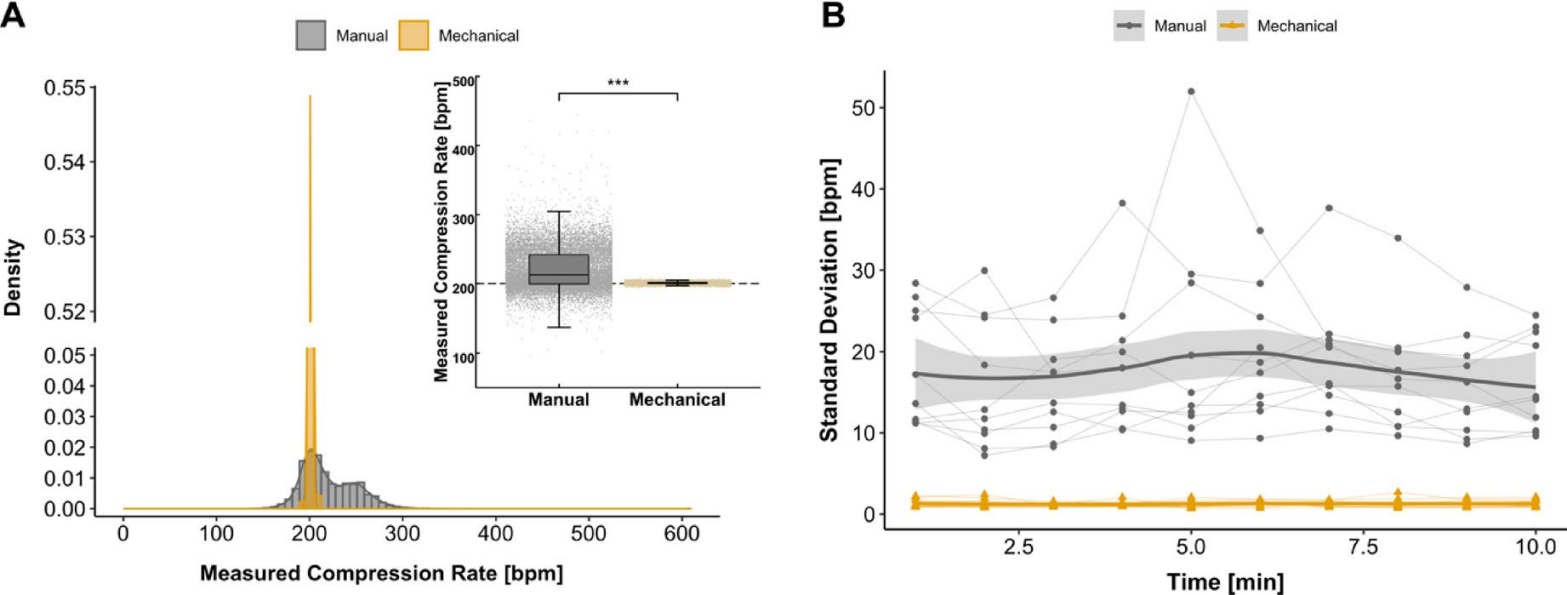
Manual versus mechanical compressions using MCD at a target compression rate of 200 bpm. (**A**) Density plot showing distribution of measured compression rates for manual and mechanical compressions at a target compression rate of 200 bpm. Internal graph showing the measured compression rates of all recorded compressions depending on type of compression. Each point represents the instantaneous compression rate between two adjacent compressions. (**B**) Standard deviation over time showing intra- and inter-provider variability at a target compression rate of 200 bpm. *p* < 0.001 for manual vs. mechanical chest compressions. *** *p* < 0.001; bpm, beats per minute.

At a target compression rate of 200 bmp, manual compressions resulted in an actual measured compression rate of 218 ± 21.0 bpm on average, in comparison to 201 ± 1.2 bmp for mechanical compressions (*p* < 0.001). As illustrated in Fig. 2A, a substantial proportion of all manual compressions out of range were conducted at an elevated compression rate (92.0%, *n* = 8993, see additional Fig. 1), with only a minor percentage of compressions being performed with a lower compression rate (8%, *n* = 777). The MCD was within the target compression range in 100% of time, in contrast to 58.8 ± 35.7% of time with manual compression (*p* < 0.001, see Table 1). At target compression rates of 200 bpm, mechanical compressions were found to be advantageous in terms of standard deviation, variance, minimal or maximal compression rate (see Table 1).

**Fig. 3:**
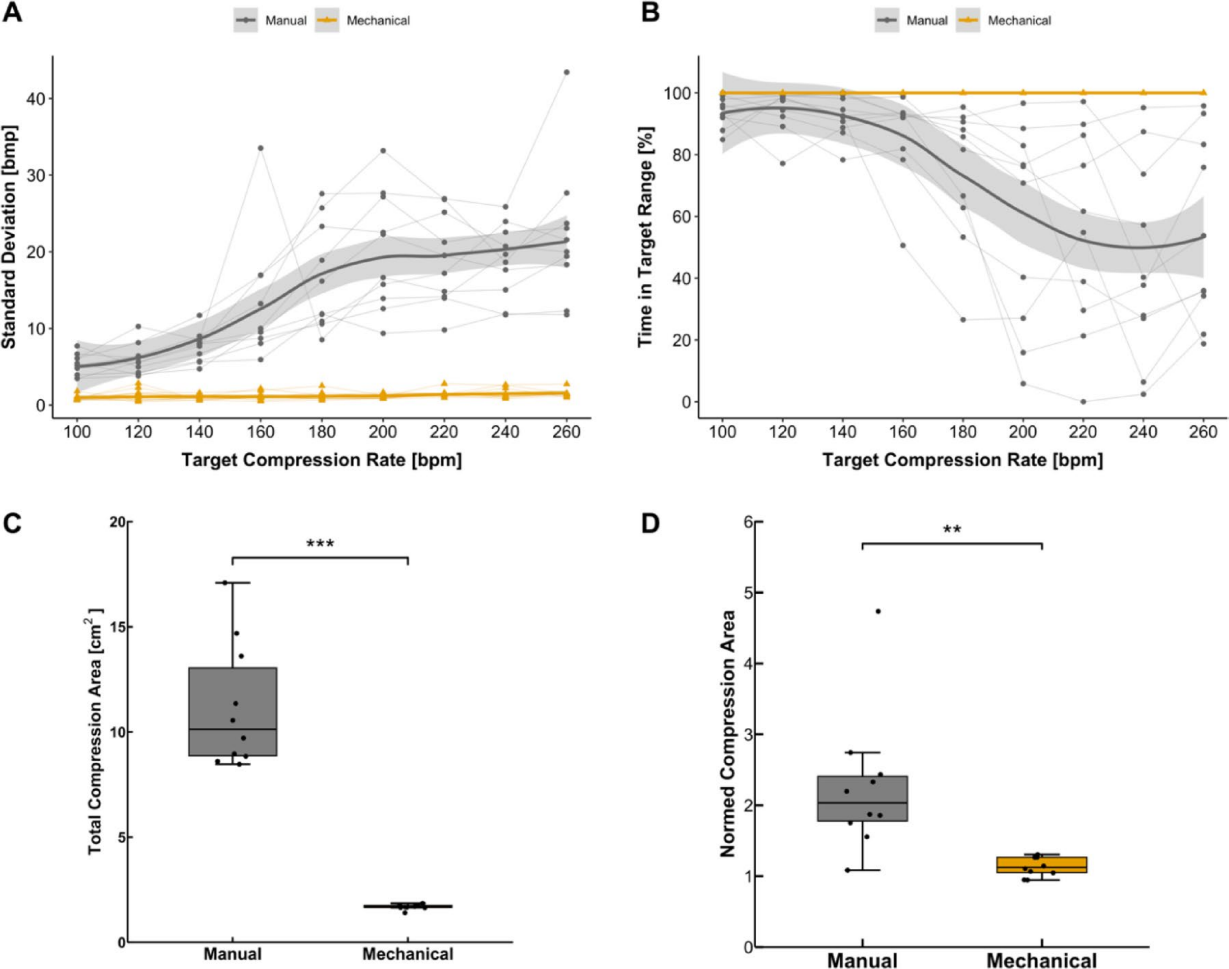
Inter-provider variability in the applied compressions and compression point in vitro. (**A**) Change in standard deviation of the actual compression rate with the chosen target compression rate. *p* < 0.001 for manual vs. mechanical chest compression for each compression rate. (**B**) Time in the target range as a function of the target rate. Target range was defined as target rate ± 10%. *p* < 0.001 for manual vs. mechanical chest compression for each compression rate. (**C**) Total area used for compression as a marker for pressure point variance. (**D**) Total area normed to different baseline compression areas of the participants’ fingers or the piston. **p < 0.01; *** *p* < 0.001; bpm, beats per minute.

Manual compressions were found to have a high degree of inter-provider variability, as shown in Figs. 2B and 3A,B. No changes over time were observed within the respective groups in the standard deviation of the actual compression rate (see Fig. 2B) or actual measured compression rate (see additional Fig. 2).

**Table 1:** Differences between manual and mechanical compressions in vitro.

	Manual compression	Mechanical compression	*p*-Value
Target compression rate [bpm]	200	200	n/a
Analyzed compressions [n]	21 650	20 098	
Mean compression rate [bpm]	218 ± 21.0	201 ± 1.2	**< 0.001**
Minimal compression rate	169 ± 26.6	198 ± 1.7	**< 0.001**
Maximal compression rate	288 ± 57.7	204 ± 1.5	**< 0.001**
Variance [bpm^2^]	369 ± 375.7	2 ± 1.3	**< 0.001**
Standard deviation [bpm]	17.6 ± 7.9	1.3 ± 0.4	**< 0.001**
Coefficient of variation [%]	8.0 ± 3.5	0.6 ± 0.2	**< 0.001**
Time in target range* [%]	58.8 ± 35.7	100 ± 0	**< 0.001**

Inter-provider variability at a constant target compression rate of 200 bpm over a period of 10 min. Parameters shown as mean ± standard deviation.

* target range equivalent to 200 bpm ± 10%, bpm, beats per minute; n, number; n/a, not applicable.

Significant values are in bold.

At lower target rates, the in vitro groups showed a higher accuracy with lower variance in SD compared to higher target compression rates (manual vs. mechanical: 100 bpm: 101 ± 5.3 bpm vs. 101 ± 0.9 bpm; 260 bpm: 287 ± 22.1 vs. 261 ± 1.5 bpm). A notable rise in SD of 2.3 bpm respectively 0.07 bpm per increase in frequency by 20 bpm (*p* < 0.01) was found in manual respectively mechanical compression, which proved a greater heterogeneity in compression rates with higher target rates (see Fig. 3A). With regard to the time in the target range, a significant reduction in time of 3.3% per increase compression rate of 10 bmp was shown (see Fig. 3B, *p* < 0.001). Using the MCD, no changes were observed by changing the target compression rates (*p* = 0.12).

Regarding the compression point, there was a significant difference in cumulative area on which compressions were performed between manual and mechanical compressions in vitro (10.8 ± 3.1 cm^2^ vs. 1.7 ± 0.1 cm^2^, *p* < 0.001) even after correction for the different contact surfaces of participants fingers or the piston (2.3 ± 0.94 vs. 1.5 ± 0.13, *p* < 0.01) (see Fig. 3, C and D).

### In vivo evaluation

**Fig. 4:**
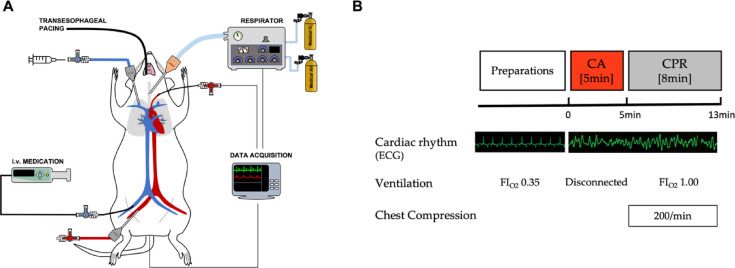
Experimental setup in the in vivo experiments. (**A**) Schematic diagram of the experimental in vivo setup. (**B**) Flow chart of the experimental procedure. i.v., intravenous; CA, cardiac arrest; Mech., mechanical; CPR, cardiopulmonary resuscitation; FI_O2_, fraction of inspired oxygen; ECG, electrocardiogram.

**Fig. 5:**
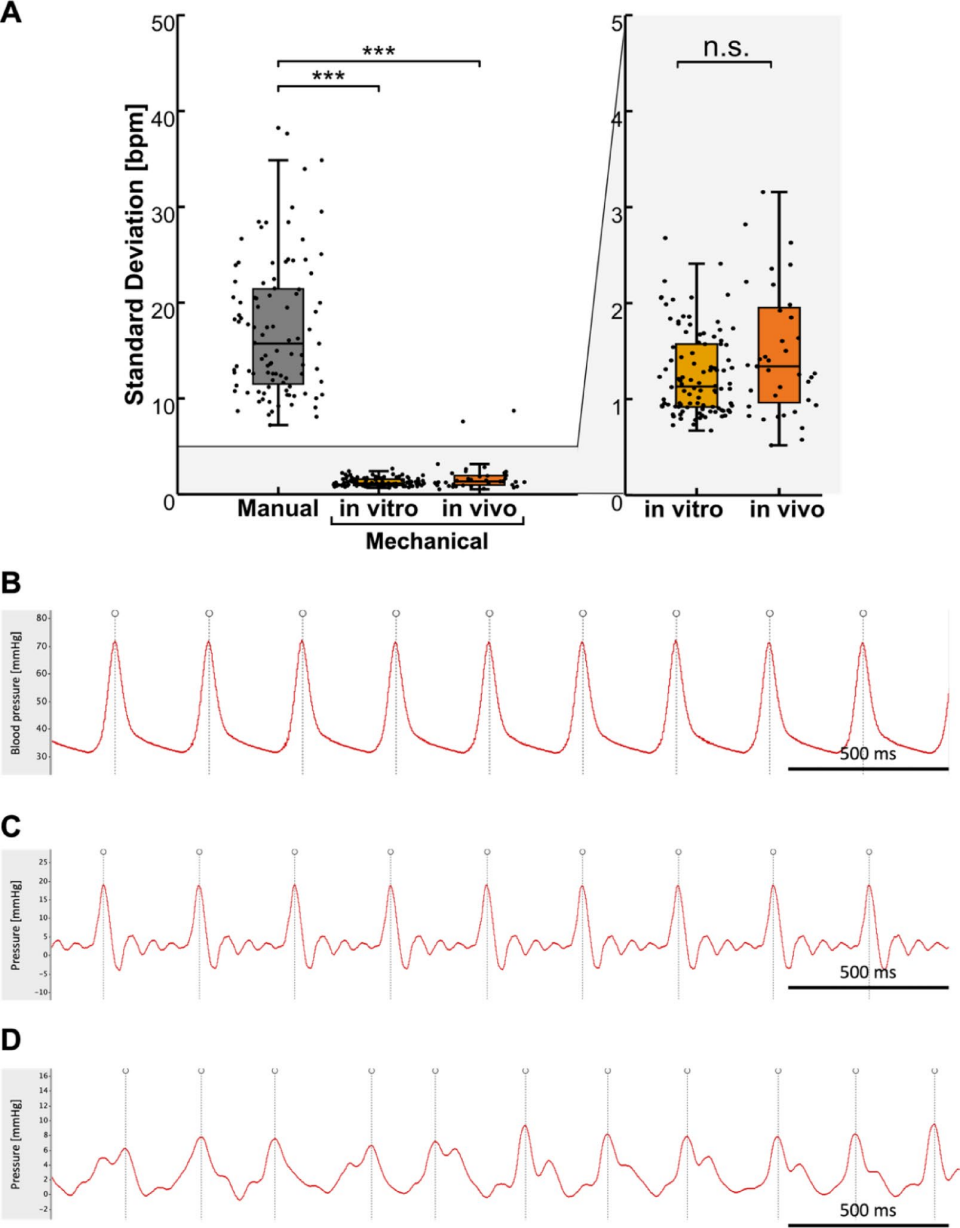
Comparison of in vitro and in vivo findings. (**A**) Mean standard deviation of actual compression rate at a target compression rate of 200 bpm in vivo and in vitro. For a more comprehensive representation of the data, a 60-second average was calculated for each instance. (**B**) Representative longitudinal course of carotid arterial blood pressure during chest compressions ongoing for 5 min using mechanical chest compression device. Bullets showing peak pressure identified using the described algorithm. (**C**) Representative pressure waveform using mechanical chest compression device in vitro at 200 bpm. (**D**) Representative pressure waveform showing manual compression in vitro at 200 bpm. *** *p* < 0.001; n.s., not significant; bpm, beats per minute.

Following proof-of-concept in our in vitro setup, a validation study in an animal model of cardiac arrest was conducted. A total of 6,917 single chest compressions were analyzed after a previous 5-minute period of global whole-body ischemia (see Fig. 4). A representative course of arterial blood pressure during chest compression is shown in Fig. 5B.

The actual compression rate using the MCD in vivo was 204 ± 3.3 bpm. As illustrated in Fig. 5, use of the novel resuscitation device resulted in the attainment of reproducible blood pressure peaks. This is also evidenced by the standard deviation of the compression frequencies, which demonstrated a notable degree of consistency using the compression aid in vivo compared to manual compressions in vitro (1.8 ± 1.7 vs. 17.6 ± 7.9 bpm, *p* < 0.001). No significant differences could be observed between the in vitro and in vivo use of the mechanical resuscitation aid (1.3 ± 0.4 vs. 1.8 ± 1.7 bpm, *p* = 0.98) (see Fig. 5). Chest compression rates were within the defined target range in 100% ± 0% of time in both, the in vitro and in vivo model.

## Discussion

This study introduces the first design of a novel MCD for performing highly standardized chest compressions on small animals. The device is intended to enable fundamental investigations on cardiac arrest and cardiopulmonary resuscitation in laboratory animal research. The application of manual chest compressions to a limited area of an animal’s chest has demonstrated to result in highly variable and non-standardized compression location, coupled with an extreme degree of variance in the applied compression rates. It could be demonstrated that the discrepancy between the actual and target compression rates in manual chest compressions increased significantly with increasing target compression rates. The present study offers an initial proposal for a straightforward design of a small animal MCD that could be rapidly integrated into existing models. The device exhibited the capacity to maintain a markedly standardized and consistent compression point and compression rate, with minimal variation, even at higher compression rates. Following comprehensive evaluation in vitro, these results were confirmed in an in vivo setup in rats.

In the field of laboratory animal research, small animal models play an indispensable role in investigating cardiac arrest and cardiopulmonary resuscitation. Such models are widely used to investigate novel therapeutic modalities, examine pathophysiological pathways, and refine existing CPR protocols^4,20,21^. A standard compression rate of 200–400 compressions per minute is often used in rat models, which is appropriate given the rat’s higher spontaneous heart rate compared to humans^9,20,22,23^. Chest compression quality is known to be a crucial predictor of successful resuscitation and imbalance between animals or groups will affect outcome measures^22,24,25^. In the Utstein-style guidelines for uniform reporting of laboratory CPR research, various American, Canadian and European professional associations, including the American Heart Association, recommend that the techniques for chest compressions used should be standardized and reported in a consistent manner^26^. They also emphasized the importance of quantifying and controlling the quality of chest compressions, and monitoring them using surrogate parameters such as coronary perfusion pressure or coronary blood flow^26^. Consequently, standardizing the execution of chest compression is of paramount importance in CPR research.

A valuable contribution to the field by Vognsen and colleagues evaluated all currently known animal models and revealed that only 11% of rodent CPR models are currently monitored for the quality of applied external chest compressions^4^. In their study, they demonstrated that in the majority of rodent studies, the selected method for closed-chest compression was not adequately reported or not specified. Of the studies that do describe the method, approximately 50% utilized manual chest compressions^4^. Our data indicates that this is accompanied by a considerable degree of variability in the quality of compressions applied. A lack of standardization at this early stage in the model may introduce confounding factors that could affect the primary endpoints or increase the variability of the generated data. The use of mechanical compression devices was described in 29% of rodent models. However, in the majority of cases, the device used was not adequately described or even validated^4^. The current heterogeneity of employed techniques precludes the possibility of comparing individual models with one another. Moreover, neglecting this source of artefact may lead to increased variability in the data and consequently, raise the number of subjects included in animal studies unnecessarily.

A major shortcoming of our current MCD design is the absence of a mechanism to regulate or monitor additional critical parameters of chest compressions, such as the compression-to-decompression ratio (CDR), duty cycle, active decompression, compression waveform, compression time, and inter-compression pause. These parameters are crucial for a full validation of a mechanical chest compression device. In contrast, the fixed flywheel and the associated regulation of compression depth via the distance between the compression piston and the thorax result in a co-dependent relationship between the compression depth and the time spend in compression/decompression as well as the duty cycle. As the compression depth increases, the time spend in compression/decompression increases, resulting in a concomitant increase in duty cycle. In preceding animal studies, a decrease in CDR was significantly correlated with lower mean aortic pressure, carotid and cerebral blood flow and end-tidal CO_2_^25^. Similar changes in hemodynamics were found by altering the compression waveform and the duty cycle^24^. The circular configuration of the flywheel in our setup yielded a fixed, unmodifiable sinusoidal compression waveform, which on the one hand leads to a high level of standardization between the individuals, but on the other hand does not allow the waveform to be adapted for further investigations. Furthermore, the prototype demonstrated a few remaining design-related deficiencies. Firstly, the mechanical separation of the two vertical axes resulted in the horizontal axis becoming jammed when the compression depth was adjusted at different speeds on the different axes. Secondly, our device enables the setting and internal monitoring of individual parameters. However, there is currently no option to export this data for further analyses and uniform reporting. We plan to address the identified deficiencies mentioned in a future development step.

Our study has several limitations. Firstly, in the in vitro setting, the measurement and evaluation of chest compression quality was limited to heterogeneity in compression rate and of the pressure point. Consequently, assessment of the generated blood flow, additional hemodynamic parameters (e.g., blood pressure, end-tidal CO_2_) and compression depth, recognized as key indicators of the quality of chest compressions in vivo, was not possible. End-tidal CO_2_, a surrogate marker for cardiac output, could not be measured in this study due to the unavailability of the necessary technical equipment for reliable capnography in small rodents. Secondly, pressure point variability could not be assessed in the in vivo model. The hypothesis that a consistent compression point may reduce tissue trauma or improve perfusion remains unproven in this study. Further research is required to determine whether a constant compression point is indeed more beneficial than a slightly varying one in terms of outcomes and potential injuries caused by mechanical resuscitation. Thirdly, mechanical and manual chest compressions produced differently shaped pressure waveforms in the chosen in vitro model. Although manual validation of the data was performed, the large number of recorded compressions makes it impossible to fully exclude the possibility that waveform differences contributed to the observed variability. Fourthly, the in vivo part of this study was performed exclusively in rats, including both sexes. Potential sex-related physiological differences therefore cannot be fully excluded. Moreover, the generalizability of these findings to other species (e.g., mice or larger animal models) remains uncertain. Finally, compression rates were analyzed directly on the same participant, with the possibility that fatigue may have contributed to the observed effects.

In conclusion, our study highlights that manual applied chest compressions are highly heterogeneous regarding the pressure point and the applied frequency. Therefore, we believe that gaining control over these parameters is urgently required for further standardization and comparability of animal CPR models. With this paper, we hope to enable other scientists to use this device and thus further standardize rodent research in the field of resuscitation.

## Conclusions

The developed MCD provides more consistent and reproduceable chest compressions than manual CPR. In accordance with the 3-R principle, we therefore consider the utilization of mechanical chest compression devices in small animal models to be advantageous and desirable.

## Supplementary Information

Below is the link to the electronic supplementary material.

Supplementary Material 1

Supplementary Material 2

Supplementary Material 3

## Data Availability

All analyzed datasets are available in anonymous form from the corresponding author upon reasonable request.
