# Supplementary material for: Development and evaluation of a mechanical chest compression device for standardized rodent cardiopulmonary resuscitation
Source: Sci Rep. 2025 Dec 8;15:43397. doi: 10.1038/s41598-025-31959-2 (PMC12689703; doi:10.1038/s41598-025-31959-2)
Supplement: Supplementary file 3 — Supplementary Material 3 [file 41598_2025_31959_MOESM3_ESM.docx]

Supplementary Information

***Additional file 1.*** *Computer aided design data and production files of the MCD. The complete CAD model as well as the individual sub-assemblies and production data are supplied in STEP format.*

**
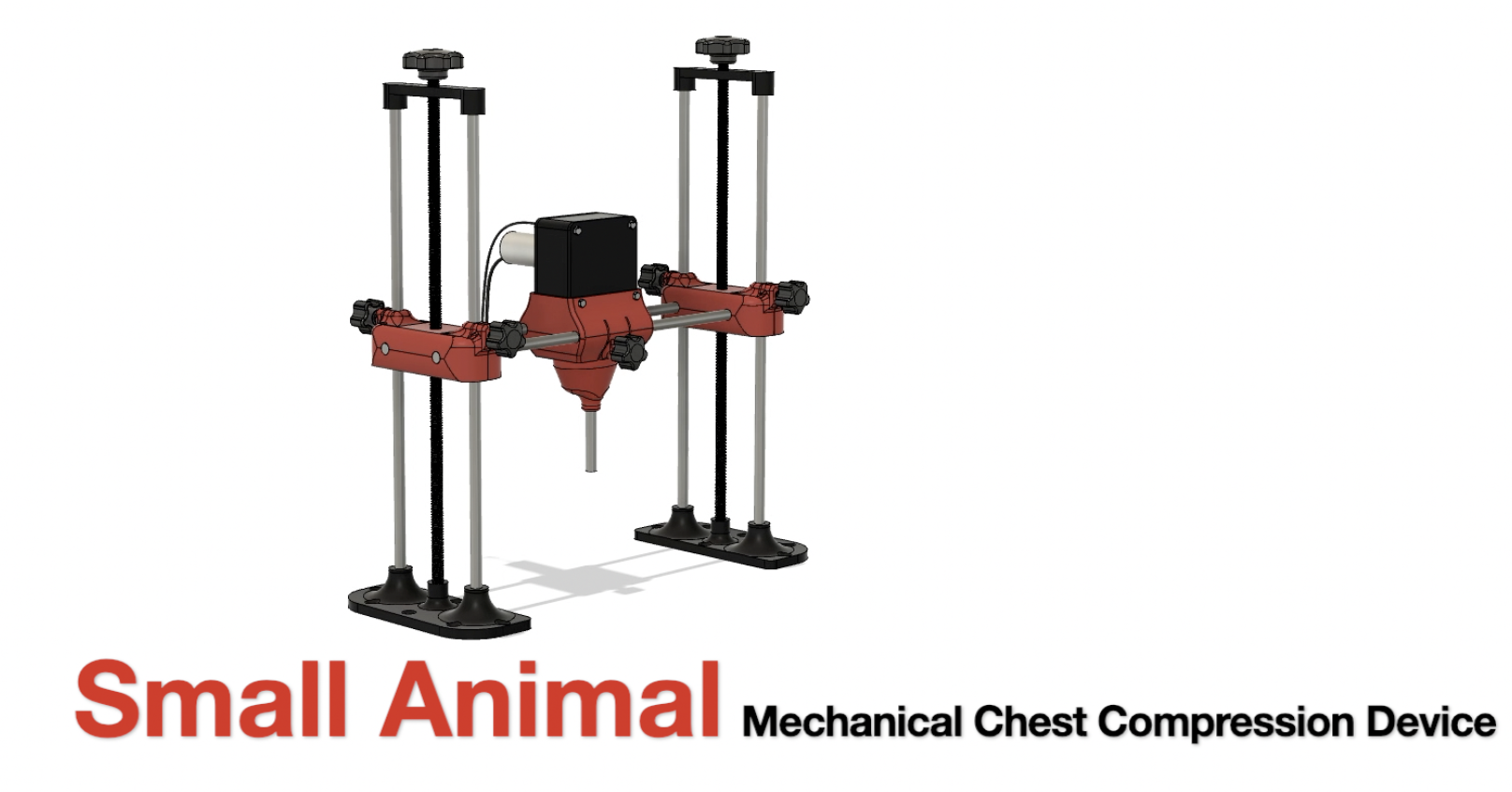
**

***Additional file 2.*** *Animation of the small animal mechanical chest compression device. Small_Animal_MCD_Design_Specification_Rev02.mp4.*

**
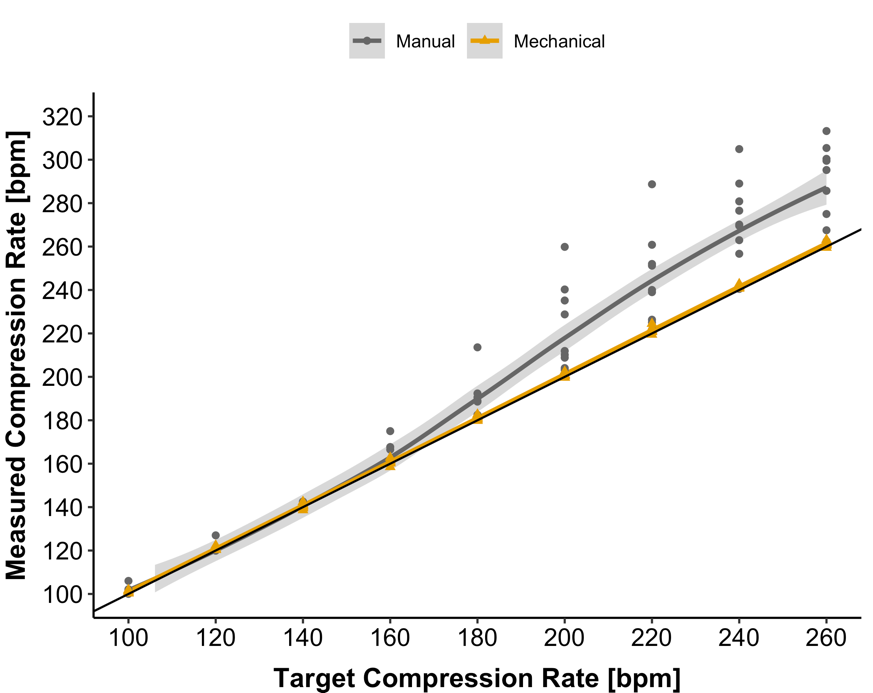
**

***Additional figure 1.*** *Mean compression rate per participant at different target rates. Different target rates (from 100 to 260 bpm) in vitro, showing an increasing deviation towards higher target compression rates in manual compression group. bpm, beats per minute.*

**
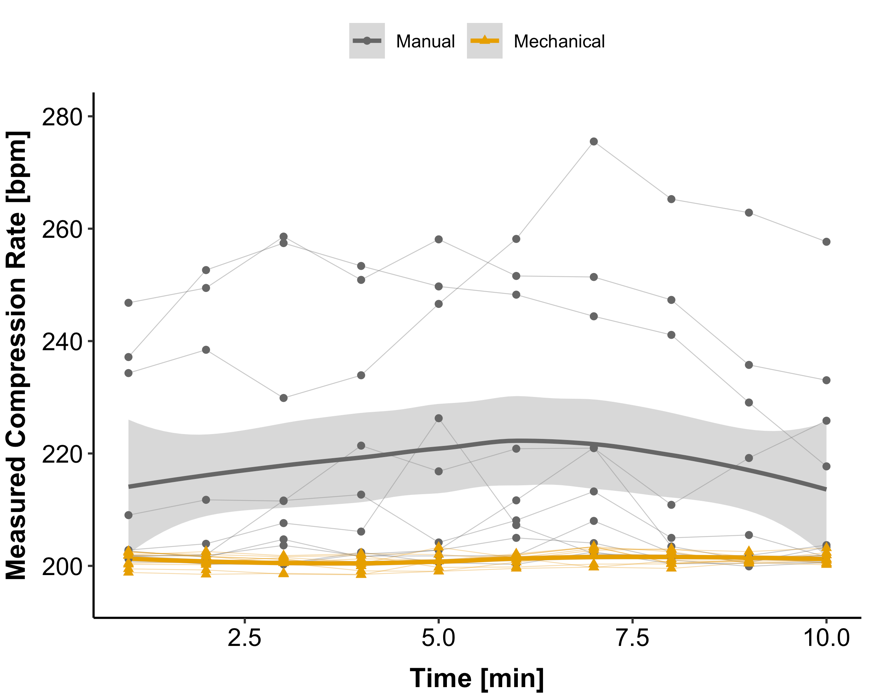
**

***Additional figure 2.*** *Mean compression rate for individual participants over acquisition period of 10 minutes. At a constant target rate of 200 bpm the measured compression rate is showing a large inter-provider variability in manual compression compared with mechanical compressions in the in vitro setting. bpm, beats per minute.*
